# Association Between Plasma microRNA Levels and Major Adverse Cardiovascular Events in Participants from an Epidemiological Cohort

**DOI:** 10.3390/ijms27209221

**Published:** 2026-10-17

**Authors:** Anna V. Kiseleva, Evgeniia A. Sotnikova, Anastasia A. Zharikova, Vladimir A. Kutsenko, Asiia E. Imaeva, Yulia A. Balanova, Anna V. Kapustina, Yuri V. Vyatkin, Maria S. Pokrovskaya, Alexandra I. Ershova, Svetlana A. Shalnova, Alexey N. Meshkov, Oxana M. Drapkina

**Affiliations:** 1 National Medical Research Center for Therapy and Preventive Medicine, Ministry of Healthcare of the Russian Federation, Petroverigsky per. 10, Bld. 3, 101000 Moscow, Russia; 2 Faculty of Bioengineering and Bioinformatics, Lomonosov Moscow State University, 1-73, Leninskie Gory, 119991 Moscow, Russia

**Keywords:** circulating microRNA, major adverse cardiovascular events, MACE, cardiovascular death, myocardial infarction, stroke, coronary artery disease, plasma

## Abstract

Circulating microRNAs (miRNAs) are promising biomarkers of cardiovascular disease (CVD) and three-point major adverse cardiovascular events (3P-MACE): CVD death, non-fatal myocardial infarction (MI), and non-fatal stroke. The associations between plasma miRNA levels and 3P-MACE in a prospective cohort from an epidemiological study were evaluated. This nested case–control study included 479 participants: 166 cases with 3P-MACE and 313 controls matched individually for sex, exact baseline age, and region of residence. Median follow-up was 10.4 years [7.0; 11.7]. Total RNA was isolated from EDTA plasma using the miRNeasy Serum/Plasma Advanced Kit. Libraries were prepared with the QIAseq miRNA UDI Library Kit and sequenced on a NextSeq 550 (1 × 75 bp). After adjustment for six and twelve preanalytical and clinical covariates, eleven and five differentially expressed (DE) miRNAs retained significant associations with 3P-MACE or CVD death, respectively. Although these findings support plasma miRNA profiles as potential MACE biomarkers, all DE miRNAs remain exploratory. Further studies in larger independent populations with cross-validation are required before integration with traditional risk markers to predict future MACE.

## 1. Introduction

Cardiovascular diseases (CVDs) are the leading cause of death worldwide, including such common conditions as coronary artery disease (CAD), hypertension, heart failure, stroke, and myocardial infarction (MI) [1]. One of the most important practical issues in modern cardiology is the accuracy of cardiovascular risk stratification, and the search for new biomarkers remains a pressing scientific challenge [2]. Molecular genetics methods offer broad opportunities to identify diseases at the earliest stages before fatal events occur and to stratify individual cardiovascular risk [3].

The microRNAs (miRNAs) are small non-coding RNAs (about 22 nucleotides) influencing gene expression on posttranscriptional level [4]. From the cell nucleus, miRNAs are transferred into plasma, circulate through the bloodstream, and are protected from degradation by incorporation into protein complexes, microvesicles, or exosomes [5].

Circulating miRNAs are potentially important diagnostic and prognostic biomarkers of various CVDs [6,7] and three-point major adverse cardiovascular events (3P-MACE) that include CVD death, non-fatal MI, and non-fatal stroke [8,9,10]. Nowadays, only a few studies showed association of circulating miRNA expression profile with cardiovascular outcomes but it is still not clear if circulating miRNAs can be used as effective predictive biomarkers in the general population [8].

The aim of this study is to evaluate the associations between plasma miRNA levels and the development of major adverse cardiovascular events (MACE) in participants of a prospective cohort from an epidemiological study.

## 2. Results

### 2.1. Study Participants Characteristics

Clinical and demographic characteristics of the participants are presented in Table 1. The case group consisted of participants who had 3P-MACE—cardiovascular death, non-fatal myocardial infarction, and non-fatal stroke. Statistically significant differences between case and control groups were found in blood pressure, high-density lipoprotein cholesterol, smoking status, arterial hypertension, and history of coronary artery disease. Two participants had a non-fatal MI or stroke followed by CVD death; therefore, the sum of individual endpoints exceeds the number of 3P-MACE cases.

### 2.2. NGS Characteristics

**Figure 1:**
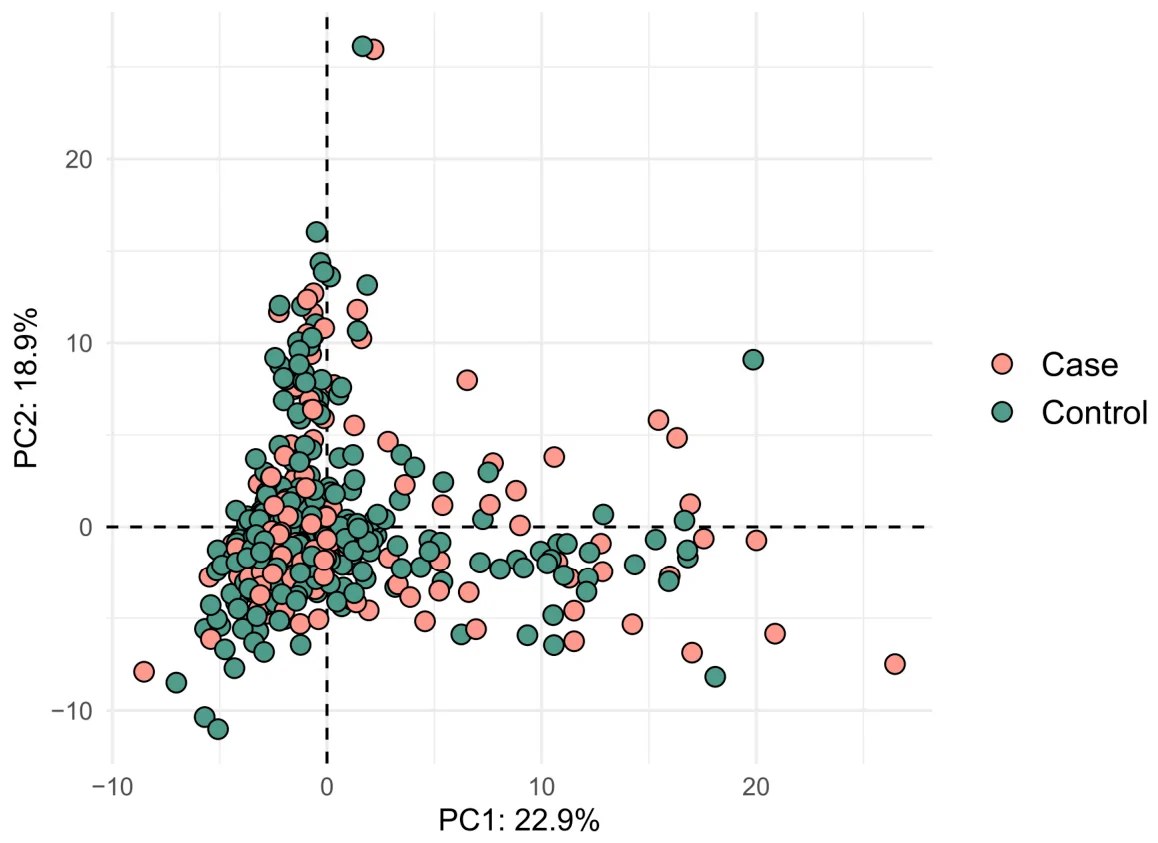
Principal component analysis of NGS data. The case group is shown in red, the control group in green.

After quality control, one control sample was excluded from further analysis. Based on human miRNA gene expression data [11], principal component analysis (PCA) showed relative homogeneity of the studied groups (Figure 1).

Comparative characteristics of the study groups are presented in Table 2. The case and control groups statistically differed significantly in plasma storage time and the percentage of spike-ins.

### 2.3. miRNAs Differential Expression

To investigate the association of miRNAs expression with CVD death, non-fatal MI and non-fatal stroke separately, participants were subdivided into four additional groups (Table 3).

**Figure 2:**
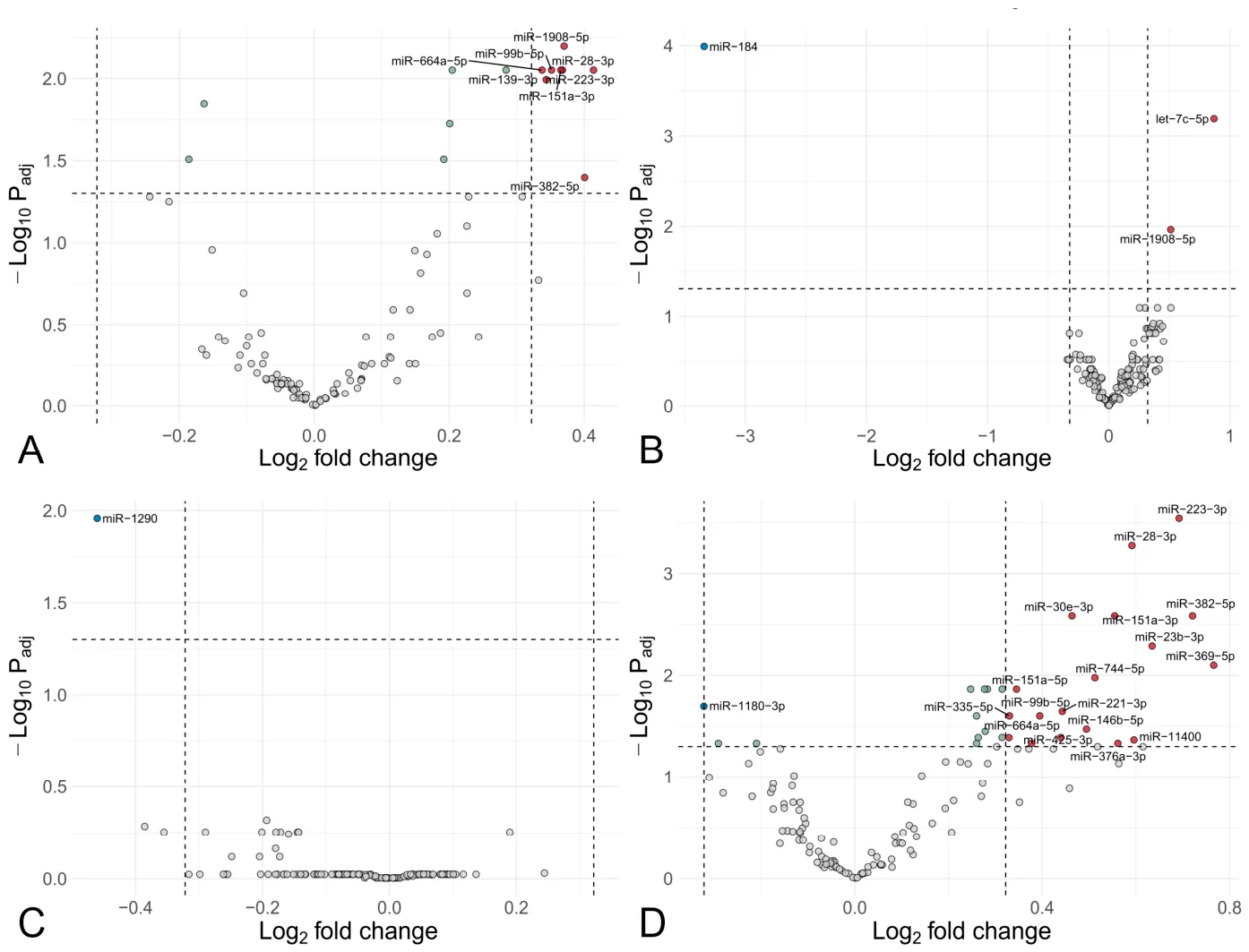
DE miRNAs in comparison groups. (**A**) 3P-MACE; (**B**) group with CVD death vs. group without CVD death; (**C**) group with fatal and non-fatal MI vs. group without fatal and non-fatal MI; (**D**) group with fatal and non-fatal stroke vs. group without fatal and non-fatal stroke. For all miRNAs in the graph, gray indicates miRNAs for which an insignificant change in expression was detected (*p*_adj_ > 0.05); blue—*p*_adj_ < 0.05 and a more than 1.25-fold decrease in expression; green—*p*_adj_ < 0.05 and a less than 1.25-fold change in expression; red—*p*_adj_ < 0.05 and a more than 1.25-fold increase in expression; *p*_adj_—*p*-values adjusted for multiple comparisons; 3P-MACE—three point major adverse cardiovascular events; CVD—cardiovascular diseases; MI—myocardial infarction.

The results of differential expression analysis are presented in Table 4, Figure 2, and Supplementary Tables S1–S5. When comparing the 3P-MACE group with the controls, 14 differentially expressed (DE) miRNAs were detected; ten of them were also found to be significantly DE between participants who had fatal and non-fatal stroke.

Overall, 37 miRNAs were found to be DE between different MACE comparison groups (Table 5). Among them, 30 were upregulated and 7 downregulated. In sensitivity analyses, we repeated the DESeq2 models with two predefined sets of covariates. Adjustment for six covariates (sex, age, plasma storage time, spike-in percentage, history of MI, and stroke) led to 11 DE miRNAs across comparisons, and the fully adjusted model with twelve covariates (sex, age, plasma storage time, spike-in percentage, history of MI and stroke, systolic blood pressure, levels of total cholesterol and high-density lipoprotein cholesterol, presence of CAD, diabetes mellitus type 2, and smoking status) identified only five DE miRNAs (Supplementary Tables S1–S5). As shown in Table 5, the impact of adjustment differed substantially by endpoint. For the composite 3P-MACE endpoint, miR-139-3p, miR-223-5p, and miR-1908-5p remained significant after full adjustment, whereas none of the miRNAs, initially associated with fatal and non-fatal stroke and with fatal and non-fatal MI, remained significant in the fully adjusted model. For CVD death, miR-184 and let-7c-5p remained after adjustment. This pattern suggests that part of the unadjusted signal was attributable to baseline history of MI or stroke, which by design was present only among cases. After this confounding was removed, the remaining non-composite endpoints, with their smaller number of events, likely had insufficient power to detect modest effect sizes. These observations emphasize that the miRNA associations, especially for individual secondary endpoints, are exploratory and require validation in larger independent cohorts. Another subanalysis showed the association with CAD for seven DE miRNAs (Supplementary Table S5, Supplementary Figure S1). This subanalysis is presented separately in Supplementary Table S5, as CAD was self-reported and was not included in the prospective part of the study.

### 2.4. miRNAs Functional Analysis

Functional enrichment analysis was performed on the eight miRNAs significantly associated with 3P-MACE (fold change > 1.25). A total of 185 putative mRNA targets were identified for these miRNAs using TargetScanHuman database [12]. Gene Ontology (GO) analysis [13] of the target gene set revealed four significantly enriched biological process categories (nuclear chromatin organization, cell junction organization, regulation of cell differentiation, positive regulation of RNA biosynthetic process). Kyoto Encyclopedia of Genes and Genomes (KEGG) pathway analysis [14] yielded one significantly enriched pathway—signaling pathways regulating pluripotency of stem cells. Notably, no enrichment was observed for pathways or GO categories related to cardiovascular disease or associated biological processes. This absence of cardiovascular-specific enrichment should be interpreted in light of the limited number of miRNAs and predicted targets analyzed, the stringency of the target prediction filters, and the possibility that circulating miRNAs may reflect systemic rather than cardiac-specific processes.

### 2.5. ROC Analysis

**Figure 3:**
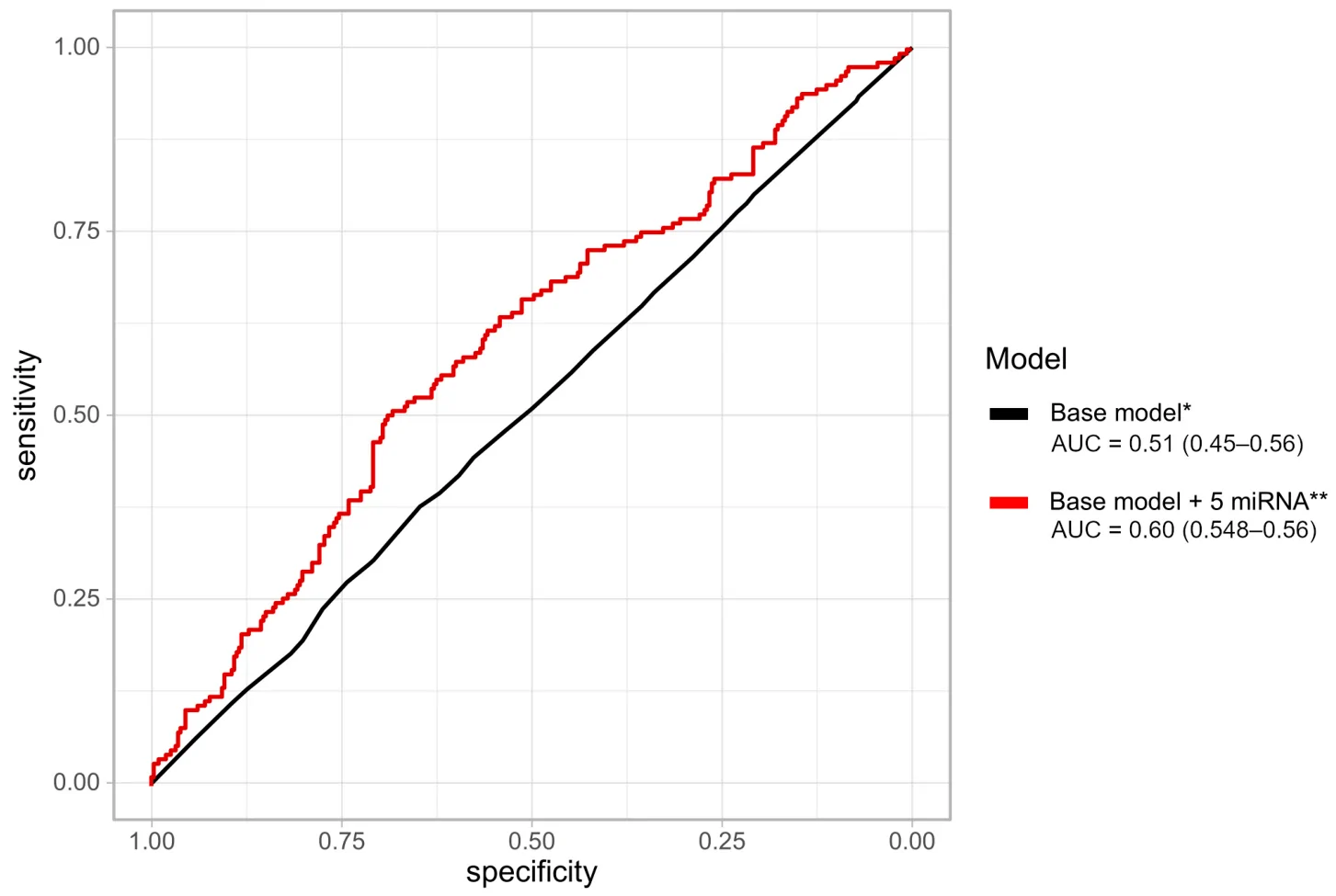
ROC curve comparison. * Model including age and sex; ** miR-139-3p, miR-320a-3p, miR-628-3p, miR-223-5p, and miR-99b-5p.

In an exploratory, post hoc ROC subanalysis, the addition of miRNA expression data was associated with a nominal improvement in the model based on age and sex (*p* = 0.014). AUC values with 95% confidence intervals for each model are shown in Figure 3. This analysis is not independently validated: the five miRNAs (miR-139-3p, miR-320a-3p, miR-628-3p, miR-223-5p, and miR-99b-5p) were selected from the list of significantly DE miRNAs without adjustment for covariates and then tested in the same dataset, without cross-validation or a separate cohort. The *p* = 0.014 is therefore a nominal, non-validated result and cannot be interpreted as evidence of discriminative or diagnostic performance. The base model has no discriminative power due to prior matching by age and sex. These findings are hypothesis-generating only and should not be relied upon.

## 3. Discussion

In this study, the association of circulating miRNAs with 3P-MACE was evaluated within the prospective epidemiological cohorts. To our knowledge, it is one of the few prospective studies that investigate the association of circulating miRNAs with documented 3P-MACE (CVD death, non-fatal MI, and non-fatal stroke).

Assessing the predictive ability of circulating miRNAs for one or more MACE has been performed earlier in a number of prospective studies [8,9,15,16,17,18,19,20,21,22,23]: CVD death—[16,17,18,21]; MI—[8,10,15,16,18,20]; stroke—[16,22]. The follow-up period in these studies was about one year [9], two years [18,20], four years [16,17,23], six years [8,10,21], and ten or more years [15,19,22]. These studies differed in the source of miRNAs; some used serum [8,15,16,17,18,19], others used EDTA plasma [9,20,21,22]; in one study, the plasma anticoagulant was not specified [23].

In the present study, the median follow-up duration was 10.4 years [7.0; 11.7] and EDTA plasma was used. This design is similar to that used in the study [22], with a mean follow-up of 9.7 years (±3.2) that also used EDTA plasma and NGS for expression analysis. Plasma levels of three miRNAs (miR-6124, miR-5196-5p, and miR-4292) were associated with the risk of stroke. None of these three miRNAs were found to be statistically significant in our study.

Of all 37 DE miRNAs found in this study, only three (miR-126-3p, miR-191-5p, and miR-451a) have been previously described in the prospective MACE studies, which investigated CVD death, non-fatal MI, and non-fatal stroke. Two miRNAs (miR-191-5p and miR-451a) were shown to be DE in serum [15] in a group with fatal and non-fatal MI as the endpoint (*n* = 96) in comparison with healthy controls (*n* = 99)—miR-191-5p was upregulated and miR-451a downregulated. In our study, miR-126-3p and miR-191-5p were upregulated and miR-451a was downregulated in plasma from participants in the fatal or non-fatal stroke group, but not in those in 3P-MACE, fatal and non-fatal MI, or CAD groups. In addition, the serum levels of miR-126-3p were shown to be significantly lower in CAD patients compared with healthy controls but were not included in the prospective analysis [8]. To our knowledge, the remaining 34 DE miRNAs identified in our study have not been previously described in prospective MACE studies, suggesting that they may represent novel candidate biomarkers and need further investigation.

The highest fold change among differentially expressed miRNAs was detected for miR-184 (fold change = −10.12; *p*_adj_ = 0.0001). It was significant in the fully adjusted twelve-covariate model (*p*_adj_ = 0.028) but not in the six-covariate model (*p*_adj_ = 0.087). This can be explained by the fact that miR-184 has the largest fold change in the entire study, and its association may be particularly sensitive to the inclusion of specific covariates. In addition, given the modest sample size and the borderline nature of the result, this may simply reflect instability around the 0.05 significance threshold. Earlier, it was shown that higher miR-184 expression levels are associated with acute MI and MACE development among patients with acute MI during a one-year follow-up [24]. Yang et al., using a rat model, found that miR-184 was downregulated following ischemic stroke and that increasing its level alleviated brain damage [25]. In another study, it was found that the expression of miR-184 was greatly increased in an in vitro model of MI [26]. Given all the conflicting information, further research is needed to validate this result.

We identified seven DE miRNAs associated with CAD, the majority of which have been previously reported in the literature [27,28,29,30,31,32]. Previously, miR-17-5p and miR-20b-5p were also found to be downregulated in CAD patients [27,28,30], while expression change in miR-20a-5p, miR-26b-5p, miR-93-5p, and let-7c-5p were in the opposite direction compared to our results [29,31]. Both studies exhibited notable differences in design compared to our own. In contrast to our work, Han et al. [31] investigated exosomal miRNAs in patients with stable CAD. Furthermore, the study by Zhong et al. [29] was limited by small group sizes (*n* = 10) and a lack of clarity regarding whether whole blood or plasma was used for RNA extraction.

In sensitivity analyses, we repeated the DESeq2 models with two predefined sets of covariates. Adjustment for six covariates (sex, age, plasma storage time, spike-in percentage, and history of MI and stroke) led to 11 DE miRNAs across comparisons, and the fully adjusted model with twelve covariates (the same six plus systolic blood pressure, total cholesterol, HDL cholesterol, CAD, type 2 diabetes, and smoking status) identified only five DE miRNAs (Supplementary Tables S1–S5). As shown in Table 5, the impact of adjustment differed markedly by endpoint. For the composite 3P-MACE endpoint, miR-139-3p, miR-223-5p, and miR-1908-5p retained significance after full adjustment, whereas none of the miRNAs initially associated with fatal and non-fatal stroke and none of those associated with fatal and non-fatal MI remained significant in the fully adjusted model. For CVD death, miR-184 and let-7c-5p persisted after adjustment. This pattern suggests that part of the unadjusted signal was attributable to baseline history of MI or stroke, which by design was present only among cases. After this confounding was removed, the remaining non-composite endpoints, with their smaller number of events, likely had insufficient power to detect modest effect sizes. These observations emphasize that the miRNA associations, especially for individual secondary endpoints, are exploratory and require validation in larger independent cohorts. Taken together, only five of the 37 DE miRNAs (approximately 14%) retained significance after full adjustment, underscoring the importance of rigorous confounder control in circulating miRNA biomarker studies.

It should be mentioned that several clinical characteristics of the studied cohort could influence circulating miRNA levels, including arterial hypertension, smoking status, and high-density lipoprotein cholesterol level. Because circulating miRNAs are rarely restricted to a single tissue type and often regulate multiple molecular pathways [33], their plasma levels may reflect contributions from multiple organs and systemic conditions rather than cardiac-specific pathology alone. Although additional subanalyses with clinical covariates showed only minor effects on our results, these associations should still be interpreted with caution. Future studies should include adjustment for systemic conditions and validation in tissue-specific contexts [34,35]. Furthermore, functional enrichment analysis of the eight miRNAs most strongly associated with 3P-MACE did not identify any cardiovascular-specific GO terms or KEGG pathways. The only significantly enriched KEGG pathway was signaling pathways regulating pluripotency of stem cells, and the enriched GO terms were related to nucleolar chromatin organization, cell junction organization, regulation of cell differentiation, and positive regulation of RNA biosynthetic process. This argues against a direct cardiovascular-specific mechanism. It can be explained by the pleiotropic, multi-tissue origin of circulating miRNAs and the limited ability of predicted-target enrichment to capture non-canonical functions, indirect effects, or cell type-specific regulatory networks. Thus, while the lack of cardiovascular pathway enrichment weakens our findings, it does not necessarily invalidate the potential utility of the found DE miRNAs as biomarkers. These associations should be considered hypothesis-generating. The stem cell pluripotency pathway, which includes TGF-β, Wnt, and PI3K-Akt signaling components, may be indirectly related to vascular repair and cardiac regeneration, but this interpretation remains speculative.

Our findings underscore the fact that various preanalytical factors such as biosample type, different miRNA detection methods, bioinformatics, and statistical analyses make cross-study comparison complicated and should be standardized due to the higher reproducibility of miRNA studies [36,37]. All these methodological issues should be resolved before circulating miRNAs can be considered as reliable biomarkers in clinical practice [15].

Our study has some limitations that need to be addressed. No risk-prediction models were fitted; the analysis evaluates associations with 3P-MACE, not risk prediction, and is hypothesis-generating. Different binary classifications (0/1) for the endpoints led to varying sample sizes of the positive and negative groups, which in turn resulted in different quality control thresholds. Stroke subtype (ischemic or hemorrhagic) was not reported. The diagnosis of CAD in our study was self-reported and not clinically verified, which could lead to an overestimated value. Controls were free of prior MI and stroke, whereas cases were not; therefore, residual confounding by prevalent cardiovascular disease cannot be fully excluded, although adjustment for baseline disease had only a modest effect on 3P-MACE but a substantial effect on the secondary endpoints. After full adjustment, only five DE miRNAs retained significance. Additionally, the significant differences in other parameters, such as plasma storage time, spike-in percentage, history of MI and stroke, systolic blood pressure, levels of total cholesterol and high-density lipoprotein cholesterol, presence of CAD, diabetes mellitus type 2, and smoking status, between cases and controls may have influenced the circulating miRNA profiles independently of cardiovascular risk. The ROC analysis was exploratory and uninformative for clinical utility: the base-model AUC of 0.51 reflects the prior matching by sex and age, and the analysis was performed after a prior miRNA selection step without correction for multiple comparisons.

## 4. Materials and Methods

### 4.1. Study Subjects

Participants were drawn from the ESSE-RF-1 and ESSE-RF-2 cohorts, both of which were part of a prospective study in 14 regions of the Russian Federation (see [38] for details). The total sample size was 22,869 individuals; the recruitment period was from 15 September 2012 to 10 November 2017. The median follow-up duration was 10.4 years [7.0; 11.7]. The information about 3P-MACE—CVD death, non-fatal MI, and non-fatal stroke—among the study participants was collected during the follow-up period. Vital status was assessed every two years in each participating region and centrally processed at the NMRC for Therapy and Preventive Medicine. Endpoints were ascertained through multiple sources, including official death registries, regional compulsory health insurance funds, medical records from hospitals for non-fatal MI and stroke and direct contact with participants or their relatives.

This study is a nested case–control study within the ESSE-RF-1 and ESSE-RF-2 prospective cohorts. Cases were 166 randomly selected participants who experienced 3P-MACE during follow-up and had available plasma samples. For each case, up to two controls were randomly selected from cohort participants who remained free of 3P-MACE during follow-up and had available plasma samples. Controls were individually matched to cases on sex, exact age at baseline and region of residence. Matching was performed at the sampling stage to ensure balance on these variables; it was not intended to create paired observations. Cases and controls were different individuals, and no control was reused for more than one case. If more than two eligible controls were available for a case, two were selected at random; if fewer than two were available, all eligible controls were included. Cases were included regardless of baseline history of MI or stroke, whereas controls were free of prior MI and stroke. This was done to preserve the population-based nature of the cohort and to avoid selection bias, since individuals with prevalent cardiovascular disease are part of the general population.

Of the 166 cases, 147 were matched to two controls and 19 to one control; no case had zero controls. The final control group comprised 313 participants. The total sample included 479 participants. Because matching was exact and the matching variables were balanced between cases and controls, the primary DESeq2 and logistic regression analyses were performed without matched-set terms.

Stroke subtype (ischemic or hemorrhagic) was not reported. CAD was determined by self-report in response to the question, “Has a doctor ever told you that you have CAD?”, and was not clinically verified.

### 4.2. Ethical Statement

The study was done according to the Declaration of Helsinki and the National Standard of the Russian Federation “Good Clinical Practice (GCP)” (GOST R 52379-2005) and was approved by the Independent Ethics Committee of the NMRC for Therapy and Preventive Medicine (protocol numbers 07-03/12 from 3 July 2012 and 03-01/17 from 18 April 2017). To comply with these regulations, as well as Article 93 of the Federal Law “On the Fundamentals of Health Protection of Citizens of the Russian Federation” (dated 21 November 2011, No. 323-FZ), the written informed consent for the processing of their personal data was obtained from all participants. To ensure participant confidentiality, all clinical, biochemical, and genetic data were de-identified and encrypted.

### 4.3. Blood and Plasma Collection

Peripheral blood was collected in K2EDTA-containing tubes. Before plasma separation, blood samples were stored at room temperature for no more than one hour and then centrifuged for 15 min at 1200× *g* at +4 °C. Plasma samples were aliquoted and immediately stored at −70 °C without freeze–thaw cycles. The detection of hemolysis was done visually for all plasma samples. For cases, the time from blood draw to the event is identical to their follow-up duration.

### 4.4. RNA Isolation

Before RNA isolation, plasma samples were thawed at 37 °C and centrifuged at 14,000× *g* for 15 min at +4 °C, then the supernatant was transferred to clean nuclease-free tubes and immediately used for the isolation of total RNA, including miRNA, with the miRNeasy Serum/Plasma Advanced Kit (Qiagen, Hilden, Germany). During isolation, QIAseq miRNA Library QC Spike-Ins (Qiagen, Hilden, Germany) were added to the plasma samples (0.5 µL of spike-in per sample). After isolation, each RNA sample was aliquoted and immediately stored at −70 °C without freeze–thaw cycles. The miRNA concentration was measured using the Qubit microRNA Assay Kit (Thermo Fisher Scientific, Waltham, MA, USA) on Qubit 4 fluorometer (Thermo Fisher Scientific, Waltham, MA, USA).

### 4.5. Next Generation Sequencing

Sequencing libraries were prepared using the QIAseq miRNA UDI Library Kit (Qiagen, Hilden, Germany). Primers and 3′ and 5′ adapters were diluted according to the manufacturer’s “10 ng” protocol version after optimization [39], using the QIAseq miRNA 96 Index Kit IL UDI-B or C. The number of cycles during library amplification was increased to 21 (instead of 19) because of very low library concentrations. The concentration of the NGS libraries was measured on a Qubit 4 fluorometer (Thermo Fisher Scientific, Waltham, MA, USA) using the Qubit 1X dsDNA HS Assay Kit (Thermo Fisher Scientific, Waltham, MA, USA). Sequencing was carried out on a NextSeq 550 platform (Illumina, San Diego, CA, USA) using a 1 × 75 bp single-end protocol.

According to the manufacturer’s protocol, when assessing the quality of prepared libraries using Bioanalyzer 2100 (Agilent, Santa Clara, CA, USA), the adapter dimer peak (~178 bp) should not exceed 25% of the height of the miRNA-containing library peak (~200 bp). Initially, we used the QIAseq miRNA 96 Index Kit IL UDI-A (96), Version 2 (REF 331905, LOT 77801720) for library preparation. In libraries prepared with this kit, the adapter dimer peak height was ~50% or more of the target peak height. However, when using the QIAseq miRNA 96 Index Kit IL UDI-B (96), Version 2 (REF 331915, LOT 77801777), the adapter dimer peak height was <25%.

**Figure 4:**
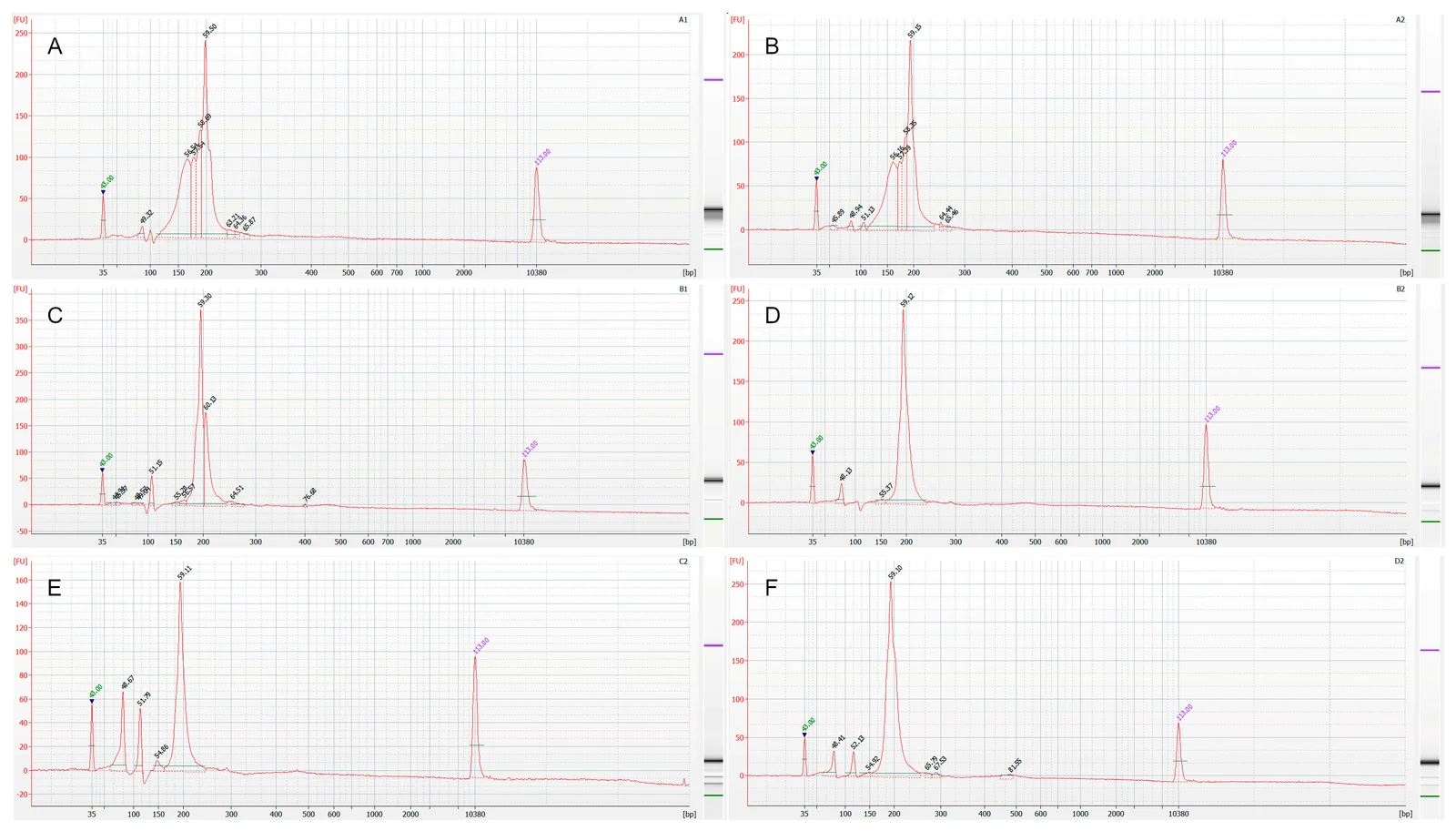
Bioanalyzer analysis of NGS libraries with different index sets. (**A**) QIAseq miRNA 96 Index Kit IL UDI-A (96); (**B**) UDI-A from QIAseq miRNA 96 Index Kit UDI A–D (384); (**C**) QIAseq miRNA 96 Index Kit IL UDI-B (96); (**D**) UDI-B from QIAseq miRNA 96 Index Kit UDI A–D (384); (**E**) UDI-C from QIAseq miRNA 96 Index Kit UDI A–D (384); (**F**) UDI-D from QIAseq miRNA 96 Index Kit UDI A–D (384).

Subsequently, RNA isolated in parallel from two aliquots of plasma obtained from a single blood sample was used to prepare libraries with the above-mentioned UDI-A (Figure 4A) and UDI-B (Figure 4C) kits, as well as with four index sets (A–D) from the QIAseq miRNA 96 Index Kit UDI A–D (384), Version 2 (REF 331977, LOT 77801867) (Figure 4B,D–F). The results of the Bioanalyzer analysis of the resulting libraries are shown in Figure 4. Both libraries, prepared using indices from set A (Figure 4A,B), had a significantly more pronounced adapter dimer peak; therefore, it was decided not to use UDI A index sets for NGS library preparation. The average height of the peak corresponding to adapter dimers in final miRNA libraries was less than 25% of the height of the miRNA peak and no additional purification to remove dimers by polyacrylamide gel electrophoresis was needed.

### 4.6. Bioinformatic Analysis

The bioinformatic analysis of small RNA sequencing data was performed using R version 4.5 following the general workflow described previously [40]. Raw single-end reads in FASTQ format were first processed for UMI removal using the UMI-tools package (version 1.1.6) [41]. Adapter trimming and length filtering were performed with cutadapt (version 5.2) [42], retaining only reads of at least 14 nucleotides in length. The filtered reads were then aligned to the mature miRNA sequences from miRBase (http://www.mirbase.org/ (accessed on 3 August 2026)) [11] using STAR (2.7.10a_alpha_220506) with the --alignEndsType EndToEnd parameter [43]. The alignment files were sorted and indexed using samtools (version 1.2.1) [44]. Gene annotation was performed using featureCounts (v2.0.1) [45] with the –primary parameter. The R package ggplot2 (version 4.0.3) [46] was used for visualization.

As part of quality control, samples with excessively large library sizes were assessed; as a result, one control sample was excluded from further analysis. For differential expression analysis, only miRNAs with more than 50 reads in at least half of the samples from the smallest experimental group were retained. miRNA differential expression between groups was evaluated using the standard tests from the DESeq2 package (version 1.42.1) [47]. Multiple testing correction was applied using the Benjamini–Hochberg procedure within the DESeq2 package (version 1.42.1) [47].

Functional analysis was performed for the revealed DE miRNAs. Predicted mRNA targets of DE miRNAs were retrieved using TargetScanHuman database [12] with filters: site type 7mer-m8 and 8mer, context score < −0.3, and predicted relative KD < 1. Functional enrichment analysis of the predicted target genes was then carried out using the GO gene annotation database [13] for the biological process category and the KEGG pathway database [14].

### 4.7. Statistical Analysis

Statistical analysis was done using R version 4.5. Continuous variables are presented as median and interquartile range: Me [Q1; Q3]; discrete variables—as absolute and relative frequencies. Continuous variables were compared using the Mann–Whitney U test, and categorical variables—using Fisher’s exact test. Effect sizes are reported as fold changes with adjusted *p*-values, and standard errors are provided in the Supplementary Materials. Given the strong dependence among the five endpoints and their overlapping participant subsets, we applied Benjamini–Hochberg correction within each endpoint rather than across all five comparisons. This approach may increase the risk of false positives across the five screens, and the results should therefore be interpreted with this limitation.

No risk-prediction models were fitted; the analysis evaluates associations with 3P-MACE, not risk prediction, and is hypothesis-generating. Two logistic regression models were constructed to evaluate the discriminative power of miRNA expression profiles: base model, including age and sex only; and full model, including age, sex, and log1p of normalized counts of selected miRNAs. We selected only five of 14 DE miRNAs with a variance inflation factor less than 2: miR-139-3p, miR-320a-3p, miR-628-3p, miR-223-5p, and miR-99b-5p. ROC curves were generated for both models using predicted probabilities. This subanalysis should be interpreted with caution, as it was conducted following a prior miRNA selection procedure and did not account for correction for multiple comparisons.

The parent epidemiological study was based on a random nested sample, and potential bias in the present analysis was addressed by adjusting for relevant confounders, including baseline cardiovascular disease, sex, age, plasma storage time, spike-in percentage, history of MI and stroke, systolic blood pressure, levels of total cholesterol and high-density lipoprotein cholesterol, presence of CAD, diabetes mellitus type 2, and smoking status. No formal power calculation was performed, as this was an exploratory discovery-phase analysis. No missing data were present for the variables included in the analysis. As a sensitivity analysis, DESeq2 was repeated with adjustment for two sets of covariates. Differences were considered statistically significant at *p* < 0.05.

## 5. Conclusions

After adjustment for six and twelve preanalytical and clinical covariates, eleven and five DE miRNAs remained significantly associated with 3P-MACE or CVD death, respectively. None of the miRNAs initially associated with fatal/non-fatal stroke or MI survived full adjustment. These findings are exploratory, derive from a single NGS dataset without independent validation, and should not be interpreted as evidence of clinical predictive utility. Further studies in larger, independent cohorts with experimental validation are necessary before these miRNAs can be considered reliable biomarkers in clinical practice and integrated with traditional risk markers to predict future MACE.

## Figures and Tables

**Table 1:** Clinical and demographic characteristics.

Variable Description	Total Sample	Case	Control	*p*-Value
Number of participants	479	166	313	
Age, years, Mean ± SD	53.50 ± 9.00	53.63 ± 8.90	53.43 ± 9.06	0.857
Sex (male), n (%)	287 (60.0)	100 (60.6)	187 (59.7)	0.922
Systolic blood pressure, mm Hg, Mean ± SD	137.82 ± 20.74	141.80 ± 21.36	135.72 ± 20.12	0.002
Diastolic blood pressure, mm Hg, Mean ± SD	87.47 ± 12.09	89.45 ± 12.30	86.43 ± 11.86	0.016
Total cholesterol, mmol/L, Mean ± SD	5.70 ± 1.22	5.70 ± 1.30	5.70 ± 1.18	0.992
HDL cholesterol, mmol/L, Mean ± SD	1.37 ± 0.34	1.32 ± 0.38	1.41 ± 0.32	0.001
LDL cholesterol, mmol/L, Mean ± SD	3.56 ± 1.05	3.55 ± 1.08	3.56 ± 1.04	0.931
Triglycerides, mmol/L, Me [Q25; Q75]	1.38 [0.91; 2.15]	1.49 [0.95; 2.25]	1.33 [0.90; 1.98]	0.063
Current smoker, n (%)	125 (26.2)	61 (37.0)	64 (20.4)	<0.001
Body mass index, kg/m^2^, Mean ± SD	29.26 ± 5.46	29.89 ± 5.99	28.94 ± 5.14	0.101
Glomerular Filtration Rate, mL/min/1.73 m^2^, Mean ± SD	88.90 ± 13.49	87.36 ± 15.64	89.71 ± 12.16	0.322
Medical history				
Statin use, n (%)	56 (12.2)	25 (15.8)	31 (10.3)	0.099
Blood pressure ≥ 140/90 mm Hg, n (%)	236 (49.5)	98 (59.4)	138 (44.2)	0.002
Total cholesterol > 5 mmol/L, n (%)	335 (70.1)	110 (66.7)	225 (71.9)	0.249
Arterial hypertension, n (%)	315 (66.5)	130 (78.8)	185 (59.9)	<0.001
History of stroke, n (%)	14 (2.9)	14 (8.5)	0 (0.0)	<0.001
History of MI, n (%)	22 (4.6)	22 (13.3)	0 (0.0)	<0.001
History of coronary artery disease, n (%)	83 (17.4)	38 (23.0)	45 (14.4)	0.022
History of type 2 diabetes mellitus, n (%)	50 (10.5)	24 (14.5)	26 (8.3)	0.041
Endpoints				
All-cause death, n (%)	72 (15.0)	54 (32.5)	18 (5.8)	
3P-MACE, n (%)	166 (34.7)	166 (100.0)	0 (0.0)	
CVD death, n (%)	54 (11.3)	54 (32.5)	0 (0.0)	
Non-fatal myocardial infarction, n (%)	48 (10.0)	48 (28.9)	0 (0.0)	
Non-fatal stroke, n (%)	66 (13.8)	66 (39.8)	0 (0.0)	

3P-MACE—three point major adverse cardiovascular events; CVD—cardiovascular diseases; HDL—high-density lipoprotein; LDL—low-density lipoprotein; Me—median; MI—myocardial infarction; SD—standard deviation; Q—quartile.

**Table 2:** Library quality and miRNA sequencing metrics.

Parameter	Case (*n* = 166)	Control (*n* = 312)	*p*-Value, Case vs. Control
Plasma storage time (−70 °C) before RNA extraction, months	103.00 [102.00; 103.00]	103.00 [103.00; 104.00]	<0.001
RNA storage time (−70 °C) before library preparation, days	14.00 [10.00; 16.75]	14.00 [11.00; 17.00]	0.42
miRNA concentration, ng/µL, Me [Q25; Q75]	0.86 [0.70; 1.05]	0.88 [0.70; 1.06]	0.35
NGS library concentration, ng/µL, Me [Q25; Q75]	1.45 [0.99; 2.47]	1.62 [1.04; 2.45]	0.86
NGS libraries storage time before sequencing, days	5.00 [5.00; 9.50]	6.00 [5.00; 10.00]	0.19
Reads per sample (library size), million, Me [Q25; Q75]	2.29 [1.89; 3.17]	2.26 [1.83; 2.81]	0.32
Spike-in, %, Me [Q25; Q75]	11.99 [7.94; 15.99]	13.90 [9.94; 18.73]	0.0011
Mapped reads, million, Me [Q25; Q75]	0.41 [0.28; 0.59]	0.40 [0.27; 0.59]	0.76
Number of expressed miRNAs (≥50 reads per miRNA), Me [Q25; Q75]	83.00 [71.00; 102.50]	82.00 [65.75; 98.00]	0.43

Me—median; NGS—next-generation sequencing; miRNA—microRNA; Q—quartile.

**Table 3:** The description of comparison groups.

Group Name	Outcome Present	Outcome Absent
3P-MACE	166	312
CVD death	54	424
Baseline, fatal and non-fatal MI	79	398
Baseline, fatal and non-fatal stroke	78	399

3P-MACE—three-point major adverse cardiovascular events; CVD—cardiovascular diseases; MI—myocardial infarction.

**Table 4:** The number of DE miRNAs associated with MACE.

Characteristic	3P-MACE	CVD Death	Baseline, Fatal and Non-Fatal MI	Baseline, Fatal and Non-Fatal Stroke
Number of DE miRNAs	14	3	1	30
Number of upregulated miRNAs	12	2	0	27
Number of downregulated miRNAs	2	1	1	3

3P-MACE—three-point major adverse cardiovascular events; CVD—cardiovascular diseases; DE—differentially expressed; MACE—major adverse cardiovascular events; MI—myocardial infarction; miRNA—microRNA.

**Table 5:** List of DE miRNAs.

miRNA, Expression Direction|Fold Change/*p*_adj_	3P-MACE	CVD Death	Baseline, Fatal and Non-Fatal MI	Baseline, Fatal and Non-Fatal Stroke
hsa-miR-23a-3p	—	—	—	up|1.20|0.047
hsa-miR-23b-3p	—	—	—	up|1.55|0.0051
hsa-miR-24-3p	up|1.14|0.031	—	—	up|1.22|0.0136
hsa-miR-28-3p	up|1.29|0.0089 ^#^	—	—	up|1.51|0.00053
hsa-miR-30e-3p	—	—	—	up|1.38|0.0026
hsa-miR-99b-5p	up|1.28|0.0089 ^#^	—	—	up|1.31|0.025
hsa-miR-126-3p	—	—	—	up|1.21|0.014
hsa-miR-126-5p	—	—	—	up|1.20|0.041
hsa-miR-128-3p	—	—	—	up|1.21|0.036
hsa-miR-139-3p	up|1.27|0.01 ^#, §^	—	—	—
hsa-miR-146b-5p	—	—	—	up|1.41|0.034
hsa-miR-151a-3p	up|1.29|0.0089 ^#^	—	—	up|1.47|0.0026
hsa-miR-151a-5p	up|1.22|0.0089 ^#^	—	—	up|1.27|0.014
hsa-miR-184	—	down|−10.12|0.0001 ^§^	—	—
hsa-miR-191-5p	—	—	—	up|1.24|0.014
hsa-miR-221-3p	—	—	—	up|1.36|0.023
hsa-miR-223-3p	up|1.33|0.0089 ^#^	—	—	up|1.62|0.00029
hsa-miR-223-5p	up|1.15|0.0089 ^#, §^	—	—	up|1.19|0.014
hsa-miR-320a-3p	down|−1.12|0.014	—	—	—
hsa-miR-320b	down|−1.14|0.031	—	—	down|−1.16|0.047
hsa-miR-335-5p	—	—	—	up|1.26|0.025
hsa-miR-340-5p	—	—	—	up|1.30|0.047
hsa-miR-361-5p	—	—	—	up|1.20|0.025
hsa-miR-369-5p	—	—	—	up|1.70|0.0079
hsa-miR-376a-3p	—	—	—	up|1.48|0.047
hsa-miR-382-5p	up|1.32|0.041	—	—	up|1.65|0.0026
hsa-miR-425-3p	—	—	—	up|1.36|0.041
hsa-miR-451a	—	—	—	down|−1.22|0.047
hsa-miR-628-3p	up|1.15|0.019 ^#^	—	—	—
hsa-miR-664a-5p	up|1.26|0.0089 ^#^	—	—	up|1.26|0.041
hsa-miR-744-5p	—	—	—	up|1.43|0.011
hsa-miR-1180-3p	—	—	—	down|−1.25|0.02
hsa-miR-1290	—	—	down|−1.38|0.011	—
hsa-miR-1301-3p	—	—	—	up|1.24|0.041
hsa-miR-1908-5p	up|1.29|0.0064 ^#, §^	up|1.43|0.011 ^#, §^	—	—
hsa-miR-11400	—	—	—	up|1.51|0.043
hsa-let-7c-5p	—	up|1.83|0.00064 ^#, §^	—	—

3P-MACE—three-point major adverse cardiovascular events; CVD—cardiovascular diseases; DE—differentially expressed; MI—myocardial infarction; miRNA—microRNA; *p*_adj_—*p*-values adjusted for multiple comparisons; ^#^—*p*_adj_ < 0.05 in subanalysis with six covariates (sex, age, plasma storage time, spike-in percentage, history of MI and stroke); ^§^—*p*_adj_ < 0.05 in subanalysis with twelve covariates (sex, age, plasma storage time, spike-in percentage, history of MI and stroke, systolic blood pressure, levels of total cholesterol and high-density lipoprotein cholesterol, presence of CAD, diabetes mellitus type 2, and smoking status). *p*_adj_ for subanalyses with six and twelve covariates are presented in Supplementary Tables S1–S5.

## Data Availability

The data used in this study, including individual information, are not publicly available due to the rules of the Ethics Committee of the National Medical Research Center for Therapy and Preventive Medicine. De-identified data can be made available upon reasonable request directed to the corresponding author, Anna Kiseleva (akiseleva@gnicpm.ru), or the Ethics Committee of the National Medical Research Center for Therapy and Preventive Medicine (phone number +74995536810, secretarynec@gnicpm.ru). Data access proposals will be reviewed and approved by the investigators, local regulatory authorities, and the Ethics Committee. Following approval, data can be transferred through a secure online platform after signing a data access agreement and a confidentiality agreement.

## Supplementary Materials

The following supporting information can be downloaded at: https://www.mdpi.com/article/10.3390/ijms27209221/s1.

## Abbreviations

The following abbreviations are used in this manuscript:

3P-MACE	Three-point major adverse cardiovascular events
CAD	Coronary artery disease
CV	Cardiovascular
CVD	Cardiovascular diseases
DE	Differentially expressed
GO	Gene Ontology
HDL	High-density lipoprotein
KEGG	Kyoto Encyclopedia of Genes and Genomes
LDL	Low-density lipoprotein
Me	Median
MI	Myocardial infarction
miRNA	microRNA
NGS	Next-generation sequencing
NMRC	National Medical Research Center
*p*_adj_	*p*-values adjusted for multiple comparisons
PCA	Principal component analysis
PCR	Polymerase chain reaction
Q	Quartile
UMI	Unique molecular identifiers
